# Nanoluciferase Reporter Zika Viruses as Tools for Assessing Infection Kinetics and Antibody Potency

**DOI:** 10.3390/v15112190

**Published:** 2023-10-31

**Authors:** Yanqun Xu, Devin Vertrees, Yong He, Sanaz Momben-Abolfath, Xiaohong Li, Yambasu A. Brewah, Dorothy E. Scott, Krishnamurthy Konduru, Maria Rios, Evi B. Struble

**Affiliations:** 1 Laboratory of Plasma Derivatives, Division of Plasma Protein Therapeutics, Office of Tissues and Advanced Therapies, Center for Biologics Evaluation and Research, U.S. Food and Drug Administration, Silver Spring, MD 20993, USA; yanqun.xu@nih.gov (Y.X.); devin.vertrees@fda.hhs.gov (D.V.); yong.he@fda.hhs.gov (Y.H.); xiaohong.li@fda.hhs.gov (X.L.); yambasu.brewah@fda.hhs.gov (Y.A.B.); dorothy.scott@fda.hhs.gov (D.E.S.); 2 Laboratory of Molecular Virology, Division of Emerging and Transfusion Transmitted Diseases, Office of Blood Research and Review, Center for Biologics Evaluation and Research, Food and Drug Administration (FDA), Silver Spring, MD 20993, USA; krishnamurthy.konduru@fda.hhs.gov (K.K.); maria.rios@fda.hhs.gov (M.R.)

**Keywords:** reporter Zika virus, nanoluciferase ZIKV, neutralization assays, bioluminescence

## Abstract

Zika virus (ZIKV) has become endemic in multiple tropical and subtropical regions and has the potential to become widespread in countries with limited prior exposure to this infection. One of the most concerning sequelae of ZIKV infection is the teratogenic effect on the developing fetus, with the mechanisms of viral spread to and across the placenta remaining largely unknown. Although vaccine trials and prophylactic or therapeutic treatments are being studied, there are no approved treatments or vaccines for ZIKV. Appropriate tests, including potency and in vivo assays to assess the safety and efficacy of these modalities, can greatly aid both the research of the pathophysiology of the infection and the development of anti-ZIKV therapeutics. Building on previous work, we tested reporter ZIKV variants that express nanoluciferase in cell culture and in vivo assays. We found that these variants can propagate in cells shown to be susceptible to the widely used clinical isolate PRVABC59, including Vero and human placenta cell lines. When used in neutralization assays with bioluminescence as readout, these variants gave rise to neutralization curves similar to those produced by PRVABC59, while being better suited for performing high-throughput assays. In addition, the engineered reporter variants can be useful research tools when used in other in vitro and in vivo assays, as we illustrated in transcytosis experiments and a pilot study in guinea pigs.

## 1. Introduction

A flavivirus with a tropism for the developing neuronal cells, Zika virus (ZIKV) is the etiologic agent for the Congenital Zika Syndrome, a constellation of signs and symptoms affecting children born to pregnant women infected during pregnancy. Common findings include fetal loss, microcephaly, parenchymal or cerebellar calcifications, ventriculomegaly, ocular dysfunction, and skeletal deformities [1]. The consequences are not only severe but also long-lasting: one in seven children aged one year or older and born to US mothers with confirmed infection during pregnancy had a birth defect or neurodevelopmental anomaly related to ZIKV infection [2]. Fetal infection and severity is higher when maternal infection occurs in the first and second trimesters [3], but negative outcomes have also been associated with third trimester infections [4]. Although at lower levels than during the 2016 outbreak, ZIKV transmission persists in many countries [5], and the risk for widespread infections exists, especially in countries naïve to such exposures. Although vaccine trials are underway [6], and prophylactic or therapeutic treatments with anti-ZIKV antibodies, including during pregnancy, have been proposed as viable options ([7], ClinicalTrials.gov (accessed on 27 October 2023) studies NCT03624946, NCT03443830), to date, there are no approved treatments or vaccines for ZIKV. Before commencing clinical trials, it is essential to assess any such products to ensure safety, to ascertain the mechanism of action, and to assign potency.

Potency of antibody products is determined in potency assays, which are quantitative evaluations of the activity linked to the primary mechanism of action. For antibody antiviral products, such as anti-ZIKV therapies, potency assays are most often neutralization assays of infectious virus in susceptible cell lines. As reviewed recently [8], neutralization assays can be performed with infectious viruses derived from clinical cases and propagated in the lab, pseudotyped virions (also called chimeric viruses), or replication-incompetent infectious particles (also called virus-like particles). For rapid and high-throughput assays, it is often desirable to use genetically modified viruses that express a reporter gene, while retaining infectivity and viral tropism. These viruses can also be used as the infectious agent for in vivo bioluminescence imaging studies which allow for the assessment of viral growth and dissemination in live animals. However, making such engineered ZIKV can be challenging due to lower infectivity and genetic instability resulting in loss of the reporter. Recently, Volkova et al. engineered and characterized a ZIKV infectious clone where the ZIKV genome was conjugated to the nanoluciferase (nLuc) gene with superior levels of expression and genetic stability [9]. The nLuc gene was chosen given its small size and strong signal [10]. We used this virus to perform infectivity and neutralization assays using bioluminescence as the readout. Assay outcomes were comparable with data obtained from PRVABC59 virus coupled with qRT-PCR readout. We also used this reporter virus for exploratory in vivo imaging studies in guinea pigs and found it recapitulated findings from other published studies performed with clinical isolates. Finally, we replaced the nLuc with teLuc gene shown to have improved spectral properties [11]. We produced teLuc-ZIKV reporter virus and tested its in vitro infection kinetics to confirm that this variant would likely prove advantageous when used in vivo.

## 2. Methods

### 2.1. Cell Lines

BeWo (human choriocarcinoma cells) clone b30 was a gift from Erik Rytting lab, University of Texas Medical Branch (UTMB, Galveston, TX, USA). Madiin Darby Canine Kidney Cell line 2 (MDCK2) transfected with human FcRn receptor (MDCK/FcRn) or the empty vector was a gift from Richard Blumberg lab, Harvard Medical School (Boston, MA USA). Jeg-3 choriocarcinoma cells (catalog number HTB-36) and Vero C1008 cells (clone E6, catalog number CRL-1586, hereafter referred to as Vero cells) were ordered from ATCC (Manassas, VA, USA).

The cells were cultured in Dulbecco’s Modified Eagle Medium (DMEM, Thermo Fisher Scientific, Waltham, MA, USA) supplemented with 10% fetal bovine serum (FBS), and Antibiotic–Antimycotic mixture (AA, Thermo Fisher), or otherwise as directed by ATCC.

### 2.2. Antibodies

ZENV14-M (mAb14) and ZENV17-M (mAb17) were purchased from Alpha Diagnostic International (San Antonio, TX, USA) and used as before [12,13]. Briefly, mAb14 is a human IgG1 anti-ZIKV envelope protein, and mAb17 is a humanized IgG1 anti-flavivirus envelope protein.

### 2.3. Viruses and Infectious Clones

ZIKV PRVABC59 was grown in Vero E6 cells and purified as previously reported [13].

**Figure 1:**
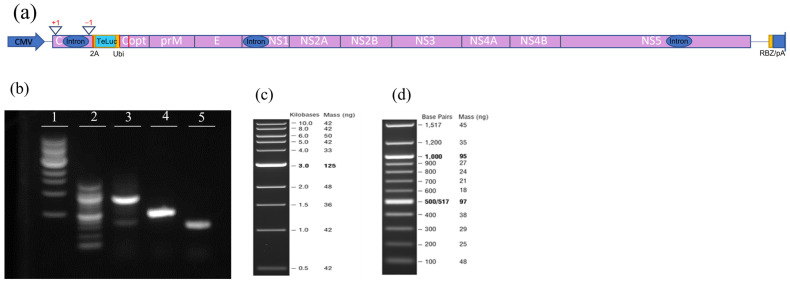
Producing an infectious clone of Zika virus (ZIKV) with teLuc, a version of the nanoluciferase gene with improved spectral properties. (**a**) Schematic representation of the genetic composition of teLuc-ZIKV infectious clone. Red vertical lines represent the position of the primers used for cloning. (**b**) Size and purity of amplicons generated during cloning. Agarose gel (1.2%) Lane 1: 1 kb DNA ladder; Lane 2: 100 bp DNA ladder; Lane 3: Amplicon 3; Lane 4: Amplicon 1; Lane5: Amplicon 2. The size and mass of the DNA ladders loaded in Lanes 1 (**c**) and 2 (**d**).

An infectious clone of ZIKV expressing the nLuc gene in the duplicated capsid gene region (nLuc-50C/FrSh), as previously described [9], was used. It features the genomic sequence of the Paraiba_01/2015 strain of ZIKV with an open reading frame shift in the first copy of the capsid gene and codon-optimizing mutations in the second copy to minimize the possibility of recombination. In addition, it contains 2A protease and ubiquitin genes to ensure the proper release of N-termini of the nLuc and capsid proteins. This infectious clone was further modified by replacing nLuc with teLuc, a mutated nanoluciferase with superior spectral properties [11]. Specifically, the teLuc gene was amplified from pcFNA3-teLuc c-myc plasmid (addgene.org, accessed on 27 October 2023) by PCR with specific primers ZIKV-2A-teLuc-Dir and teLuc-Ubi-Rev, to produce amplicon 1. Also, using the nLuc-50C/FrSh as the template and teLuc-Ubi-Dir and ZIKV-Mlu-Rev as primers, we produced amplicon 2 containing the adjacent fragment of the plasmid including the ubiquitin gene and MluI restriction site (the primers and the lengths of amplicons 1 and 2 are shown in Table 1). Amplicons 1 and 2 were purified and joined via PCR using ZIKV-2A-teLuc-Dir and ZIKV-Mlu-Rev primers to produce amplicon 3 with a length of 848 base pairs. A schematic of the genetic composition of the teLuc-ZIKV infectious clone, and the size and purity of amplicons 1–3 are shown in Figure 1.

Amplicon 3 was then inserted into the ZIKV infectious clone using XmaI, MluI, and NotI restriction sites, ligation was performed with T4 DNA ligase (Agilent Technologies, Santa Clara, CA, USA), and the mixture transformed into *E. coli* MC1061 by heat shock using standard protocols (detailed protocols are available by request). A single colony was transferred into 100 mL LB media for growing and plasmid was purified using EndoFree Plasmid Maxi Kit (Qiagen, Hilden, Germany). Plasmid (2.0 µg) was transfected into 3 × 10^6^ Vero cells suspended in 100 µL 4D-Nucleofector™ X media (Lonza Bioscience, Walkersville, MD, USA), via electroporation with the 4D-Nucleofector™ X Unit (Lonza), then transferred onto a 75 cm^2^ flask following the manufacturer’s instructions.

The nLuc- and teLuc-ZIKV reporter viruses for in vitro assays were propagated in Vero cells cultured for up to seven days then harvested as previously described [12,13]. Briefly, PEG 8000 was added to the cell culture supernatant at a concentration of 8%, the mixture was incubated overnight at 4 °C then centrifuged at 14,000× *g* for 30 min. The pellet was added to a 25% sucrose cushion and ultracentrifuged at 20,000× *g* for 1.5 h at 4 °C, then the viral particles were resuspended in DMEM with 5% FBS and stored at −20 °C.

The nLuc-ZIKV reporter virus used for live imaging was produced in C6/36 cells, purified, and titrated by focus-forming assay (FFA) as previously described [9].

### 2.4. Assessment of ZIKV Infectivity and Antibody Mediated Neutralization

Suspensions of MDCK/FcRn, MDCK/vector, Vero, BeWo or Jeg-3 cells were seeded in a flat-bottom 96-well plate and incubated at 37 °C overnight to reach 75–90% confluency. The following day, media were replaced with either nLuc- or teLuc-ZIKV alone or as a mixture with antibodies, depending on the assay being performed.

To assess kinetics of viral growth, dilutions of nLuc-ZIKV were prepared using DMEM supplemented with 2% FBS at three levels, i.e., 1:1000, 1:3000 and 1:10,000 and added to Vero cells in quadruplicates; cells with no virus served as the control. At each time point the media and cells were collected and assayed separately for luciferase activity using Nano-Glo^®^ Luciferase Assay System (Promega, Madison, WI, USA). For this, NanoGlo reagent was prepared by mixing substrate with buffer as directed. Then, 50 µL cell culture medium from each well was transferred onto a Corning Costar 96-well flat bottom white plate (MilliporeSigma, Burlington, MA, USA). When ready to record, 50 µL of NanoGlo reagent was added onto each well, the plate was incubated for 3 min then bioluminescence in the cell culture media was recorded using the Tecan Spark^®^ plate reader (Tecan U.S. Inc., Morrisville, NC, USA). For intracellular luciferase activity, all the cell culture wells were aspirated, washed with 200 µL PBS then 50 µL water and 50 µL NanoGlo reagent were added to each well. The plate was incubated at room temperature for 3 min, the contents transferred to a clean 96-well white plate and the intracellular bioluminescence recorded. The time points assessed were 24, 48, 72, 96 and 120 h post infection.

For titration assays, Vero cells were inoculated with 10-fold serial dilutions of either nLuc- or teLuc-ZIKV preparations in 2% FBS DMEM in quadruplicates for each dilution. Cells were cultured for two days, then media was removed, cells washed three times with PBS, and luciferase activity measured. Given that nLuc-ZIKV is not cytopathic in Vero cells [9], an apparent 50% tissue culture infectious dose (TCID_50_) titer was calculated using a Spearman–Kärber algorithm [14] and luciferase activity to detect infection, compared to the traditional TCID_50_ method which relies on the cytopathic effect as the indicator of infection. Specifically, wells exhibiting bioluminescent signals 10 times higher than that in media-only background were scored as positive for infection and the formula from the calculator was applied to derive TCID_50_.

For infectivity kinetics in different cell line assays, nLuc- or teLuc-ZIKV preparations were diluted 3000 times using DMEM supplemented with 2% FBS and added in four or more replicates to 75–90% confluent Vero, BeWo or Jeg-3 cells in 96-well plates; mock infection served as control. For neutralization assays, mAb14 or mAb17 antibodies were serially diluted, mixed with nLuc-ZIKV (diluted 3000 times), incubated for one hour at 37 °C, then used to inoculate 75–90% confluent Vero cells. Eight repeats for each dilution were used. The next day (neutralization assay) or at three time points (24-, 48- and 72 h post-infection, infectivity assay), media were removed, cells were washed with PBS and assessed for the presence of infection using Nano-Glo^®^ assay system (Promega) as described above. Focus-forming assay (FFA) was also performed to titer the viruses using a classical method as previously described [9].

### 2.5. Assessment of Transcytosis of Immune Complexes (IC) across Epithelial Cell Layers

Transcytosis assays were performed as previously described [13] with modifications. Briefly, single-cell suspensions of MDCK/vector, MDCK/FcRn, BeWo, or Jeg-3 cells in 200 µL DMEM 10% FBS were added onto trans-well semi-permeable membranes (6.5 mm insert, 0.4 µm pore size, 0.33 cm^2^ cell growth area) placed into Corning 24 well tissue culture plates (MilliporeSigma); 600 µL of media was added outside the trans-well. Cell growth was monitored by measuring trans epithelial electrical resistance (TEER) via EVOM2 voltohmmeter (World Precision Instruments, Sarasota, FL, USA). When TEER reached 200–300 Ohm, the transcytosis experiment using nLuc-ZIKV alone or as IC with antibodies (mAb) was performed. For this, the media were changed to DMEM supplemented with 2% FBS (200 µL inside and outside the trans-well), then 0.5 µL undiluted nLuc-ZIKV alone or as-preformed IC with 10 µg/mL anti-ZIKV mAb14 were added inside each trans-well. There were 3–6 replicates for each experiment; two independent experiments were performed. After incubation for 90 min at 37 °C, the media in the basolateral chamber were collected and transferred onto a 96-well plate containing 75–90% confluent Vero or BeWo cells to assess for the presence of infectious virus. The cells were kept in culture for two days then luciferase activity was assayed using the Nano-Glo^®^ assay system. To ascertain confluency of cells lining trans-wells, a Lucifer Yellow Lithium salt (Biotium, San Francisco, CA, USA permeability test was performed at the conclusion of the transcytosis experiment. For this, 0.2 mM Lucifer Yellow was added inside the trans-well for 60 min, then the fluorescence signal was measured in the output well, and the permeability (the ratio of fluorescent signal between the sample and the input solution) was calculated. Data from wells with permeability higher than 5% were excluded from analysis.

### 2.6. In Vivo Bioluminescence Imaging (BLI)

#### Animals

The animal protocol and procedures within this study were approved by the FDA Institutional Animal Care and Use Committee (ASP 2017-14, initial submission approval on 5 June 2017) and all methods were performed in accordance with the relevant guidelines and regulations. Young female Hartley (Crl:HA) guinea pigs, weighing 240–260 g and aged 3–4 weeks, were purchased from a commercial source (Charles River Labs, Wilmington, MA, USA). The nLuc-ZIKV reporter virus was administered subcutaneously (SC) at the scruff of the neck, at a concentration of 10^7^ focus-forming units (FFU) in 130 µL in n = 6 animals; n = 4 animals served as sham controls and received saline at the same volume. Proliferation of the infection was evaluated using an IVIS Spectrum live imaging instrument (PerkinElmer, Hopkinton, MA, USA) while under isoflurane anesthesia and 15–20 min following intra peritoneal (IP) administration of 4.2 µmole PBI 6059 substrate (Promega, available through an MTA). Images of the bioluminescent signal were collected on post-inoculation days (PID) 1, 3, and 5. Images were analyzed using Living Image^®^ version 4.7.4 software (PerkinElmer, Waltham, MA, USA). Specifically, a region of interest (ROI) that encompassed the entire body of the animal was drawn over the bioluminescent image. Two related parameters, total flux and the average radiance over the ROI, were measured/calculated for each test and control animal in every experimental day. Total flux is the number of photons per second (p/s) over the total ROI, whereas average radiance is a normalized parameter defined as photons emitted by one centimeter square of animal tissue into one steradian solid angle and has units of photons/second/cm^2^/steradian (p/s/cm^2^/sr).

### 2.7. Statistical Analysis

Data were plotted using GraphPad Prism 9 and two-sided Student *t*-tests were used to assess the differences between groups; *p* < 0.05 was considered significant.

## 3. Results

### 3.1. Assessment of Infectivity of ZIKV Infectious Clones Containing Luciferase Reporters

**Figure 2:**
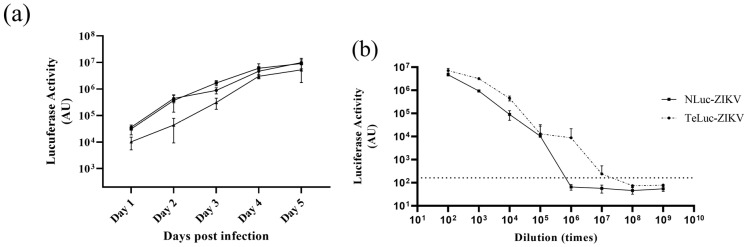
Titration and infection kinetics of reporter ZIKV in Vero cells. (**a**) Kinetics of viral growth for nLuc-ZIKV in Vero cells at three different dilutions, 1:10^3^ (●), 1:3 × 10^3^ (■), and 1:10^4^ (▲), represented as luminescence arbitrary units (AU). Means of four replicates and error bars representing standard deviations are shown. The experiment was performed once. (**b**) Titration of nLuc- and teLuc-ZIKV in Vero cells. Serial dilutions of teLuc- and nLuc-ZIKV in quadruplicates were added onto Vero cells for 48 h, and then media removed, and luciferase activity measured. Means from four repeats with error bars representing standard deviations are shown.

We produced and assayed infectious clones of ZIKV expressing nLuc [9] and teLuc genes, the latter a version of nLuc with improved spectral properties [11], cloned as described in the Section 2 (Figure 1). Both nLuc- and teLuc-ZIKV were produced in Vero cells, concentrated, and then tested for infectivity. First, three dilutions of nLuc-ZIKV were used to infect Vero cells; viral growth kinetics were assessed by measuring the secreted and intracellular bioluminescence signal (Figure 2a, only the signal measured intracellularly is shown) for up to five days post-infection. As expected, at all dilutions and time points, the intracellular signal was higher than that secreted, given that the reporter enzyme is processed and released intracellularly. A similar pattern has been seen for other virally encoded proteins for various clinical and laboratory ZIKV isolates [15].

Both nLuc-ZIKV and teLuc-ZIKV were titrated (Figure 2b) by measuring intracellular nanoluciferase signal 48 h post-infection to calculate an apparent TCID_50_ titer using a Spearman–Kärber algorithm [14]. Wells exhibiting bioluminescent signals 10 times higher than that in the sham (media-only) background (Figure 2b, broken line parallel to the x-axis) were scored as positive for infection. Applying the Spearman–Kärber algorithm resulted in a TCID_50_ of 2 × 10^7^ and 3 × 10^6^ doses/mL for teLuc- and nLuc-ZIKV, respectively. In addition, the FFA method, which relies on the quantification of ZIKV envelope glycoprotein by immunostaining, was performed and resulted in similar titers of 3 × 10^5^ and 4.5 × 10^5^ FFU/mL for teLuc- and nLuc-ZIKV, respectively. The differences between apparent TCID_50_ and FFA titers are likely due to differences in processing and production of luciferase versus mature, properly folded envelope glycoprotein.

**Figure 3:**
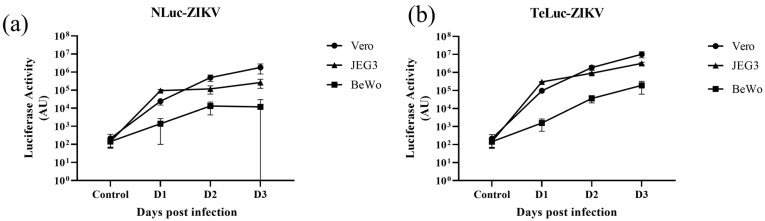
A comparison of the infectivity of two nanoluciferase ZIKV reporter viruses (**a**) nLuc-ZIKV, (**b**) teLuc-ZIKV in three different cell lines (Vero, JEG3 and BeWo). Similar dilutions of each variant were added onto 75–90% confluent Vero, JEG3 or BeWo cells. Each data point represents the average of eight repeats; error bars representing standard deviations are shown.

Next, we compared the infectivity kinetics of nLuc- and teLuc-ZIKV in Vero, BeWo, and Jeg-3 cells when inoculated at similar dilution (Figure 3). We found that the virus propagates in all these cell lines, with the highest luciferase activity produced in Vero, then Jeg-3 cells (Figure 3a). It can be noted that a similar trend can be seen with teLuc-ZIKV (Figure 3b).

### 3.2. Antibody Neutralization, Infection of Cells Overexpressing FcRn and Transcytosis of nLuc-ZIKV across Epithelial Cell Layers

**Figure 4:**
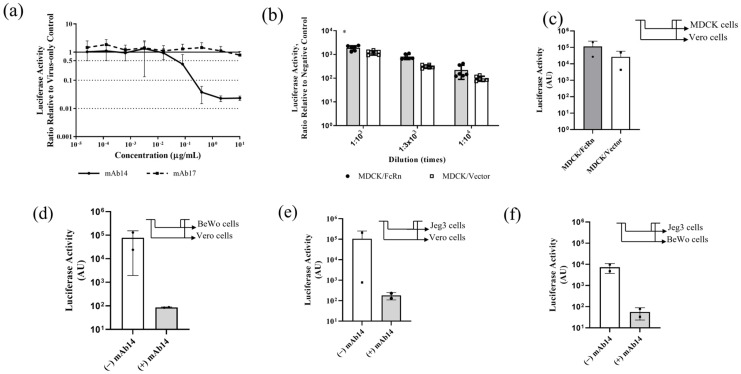
In vitro applications of nLuc-ZIKV reporter variant. (**a**) Neutralization of nLuc-ZIKV by monoclonal antibodies in Vero cells. Anti-ZIKV (mAb14, line) and anti-flavivirus (mAb17, broken line) monoclonal antibodies were serially diluted, mixed with nLuc-ZIKV then used to inoculate Vero cells. The infection was quantified using bioluminescence readout and the ratio with the no-antibody controls was plotted as a function of antibody concentration. Each data point represents averages from eight inoculations. Error bars representing standard deviations are shown. (**b**) Assessing the susceptibility to nLuc-ZIKV infection in cells that overexpress FcRn versus those that do not. MDCK/FcRn cells were more susceptible to nLuc-ZIKV infection than control cells that express an empty vector, recapitulating the findings seen with PRVABC59 strain [12]. Three different dilutions and up to eight replicates per dilution were used for each cell line. Differences are statistically significant by Student *t*-test (*p* = 0.036). (**c**) Evaluating transcytosis of infectious nLuc-ZIKV particles through a semipermeable membrane supporting confluent monolayers of MDCK/FcRn or control cells. Infectious viruses were quantified after transferring the contents of the basal chamber onto Vero cells (shown in the inset panel) and quantifying the infection using bioluminescence readout. Up to six replicates per experiment and cell line were used; the experiment was performed twice. The error bars are outside the lower limit of the y-axis. (**d**–**f**) Evaluating transcytosis through placenta trophoblast cells of infectious nLuc-ZIKV particles alone or as immune complexes (IC) with neutralizing antibody. Either BeWo (**d**) or Jeg-3 (**e**,**f**) cells were grown onto semi-permeable trans-well membranes and nLuc-ZIKV alone or as preformed immune complexes with 10 µg/mL anti-ZIKV antibody mAb14 was added inside the trans-well. The contents of the basolateral chamber were transferred onto Vero or BeWo cells as indicated in the inset schematic (top right corner of each panel). Addition of 10 µg/mL mAb14 reduces the infectivity in Vero or BeWo cells. Up to six replicates per experiment/cell line were used; each experiment was performed twice. In panel (**e**)/(−) IgG column, the error bars are outside the lower limit for *y*-axis. * *p* < 0.05.

We used nLuc-ZIKV and bioluminescence readout to assess the neutralization activities of mAb14 and mAb17 (Figure 4a), two monoclonal antibodies we previously characterized with PRVABC59 virus and PCR readout [12]. The neutralization curves from these experiments are similar, despite the differences in ZIKV isolates (PRVABC59 versus Paraiba) and assay readout (PCR and nanoluciferase activity, respectively).

We recently reported that MDCK cells that overexpress FcRn are more susceptible to PRVABC59 ZIKV infection [12]. We recapitulated this finding using the nLuc-ZIKV reporter virus. At three different dilutions, nLuc-ZIKV produces more luciferase activity in MDCK/FcRn cells (Figure 4b, shaded bars) compared to control MDCK cells that do not overexpress human FcRn (Figure 4b, clear bars). This result was statistically significant with paired two-tailed Student *t*-test (*p* = 0.036).

We then used nLuc-ZIKV reporter virus preparation to assess the transcytosis of ZIKV or ZIKV-antibody immune complexes through confluent layers of MDCK, BeWo and Jeg-3 cell lines grown on semi-permeable membranes. For this, the virus alone or as-preformed IC with anti-ZIKV mAb14 was added to the apical chamber of the trans-well for 90 min. To detect infectious virus, the contents of the output chamber were transferred to Vero or BeWo cells. Up to six replicates were included in each experiment and the experiment was repeated two independent times. We found that infectious nLuc-ZIKV transferred through all three confluent epithelial cell layers tested. Specifically, nLuc-ZIKV capable of propagating in Vero cells was found in the output chamber of trans-wells containing MDCK/FcRn and MDCK control cells (Figure 4c). Similarly, confluent human trophoblast BeWo and Jeg-3 cells were permissive to nLuc-ZIKV that was infectious to Vero (Figure 4d and Figure 4e, respectively, clear bars) and BeWo cells (Figure 4f, clear bar). Adding anti-ZIKV mAb14, decreased infection irrespective of the cells used to form the confluent layer or to detect the infection (Figure 4d–f, shaded bars). However, these findings were not statistically significant, due to the large variability observed in this assay. In addition, no antibody-mediated enhancement of infection was seen under the conditions of this study.

### 3.3. In Vivo Bioluminescent Imaging (BLI) with nLuc-ZIKV

**Figure 5:**
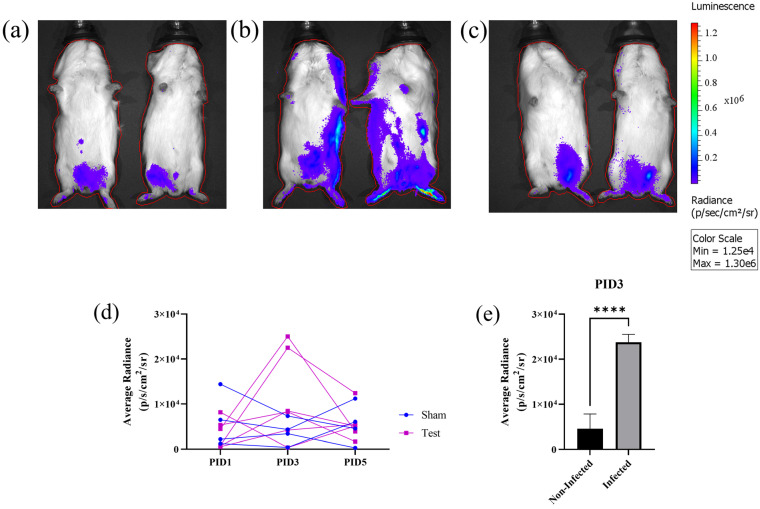
Use of nLuc-ZIKV for in vivo bioluminescence imaging (BLI). (**a**–**c**) Representative whole-body images of bioluminescent signals in control and inoculated guinea pigs on post-inoculation day (PID) 3. (**a**) Sham-inoculated, (**b**) nLuc-ZIKV-inoculated and infected, and (**c**) nLuc-ZIKV-inoculated but non-infected guinea pigs. Red line outlining each animal represents the region of interest. (**d**) Kinetics of bioluminescent signals in n = 4 sham and n = 6 nLuc-ZIKV-inoculated juvenile guinea pigs. Two out of six nLuc-ZIKV-inoculated guinea pigs have higher signal on PID 3, compared to either other animals or time points. (**e**) A comparison of bioluminescence on PID 3 for the two inoculated and infected animals shown on panel (**b**) with the signal from the control and other inoculated animals, the differences are statistically significant. **** *p* < 0.0001.

Finally, we used the nLuc-ZIKV reporter virus to assess infection kinetics and proliferation in live animals using bioluminescent imaging (BLI). Either viral preparation (10^7^ FFU/animal) or diluent buffer saline (sham) was administered subcutaneously to n = 6 and n = 4 juvenile Hartley guinea pigs, respectively. Nanoluciferase activity was assessed by whole-body bioluminescence on post-infection day (PID) 1, 3, and 5, following IP administration of substrate. We found that all animals inoculated with sham preparation had a low luminescence signal at all time points, as evidenced by the small area with pixels of low radiance (Figure 5a, colored dark blue, representative images are shown). This area coincides with the site of IP administration of the substrate, and it is likely due to the autoluminescence of the substrate. On the other hand, on PID3, 2 out of 6 animals inoculated with viral preparation had both larger areas with dark blue pixels, extending beyond the site of administration, and pixels with higher radiance as demonstrated by regions colored in brighter shades (Figure 5b). However, the other 4 out of 6 guinea pigs inoculated with the viral preparation looked similar to the sham-inoculated animals, a representative image is shown (Figure 5c). To quantify the signal, an ROI that encompassed the entire body of the animal was defined (Figure 5a–c, red contour around each guinea pig) and the total flux expressed as photons per second (p/s) and the average radiance (p/s/cm^2^/sr) was calculated for every experimental day. Both parameters have a similar time dependence, confirming the qualitative results in Figure 5a–c (Figure 5d, shown is the average radiance only). Although there was no statistical difference in the flux or radiance of infected versus sham animals, a comparison of the subset of the animals that exhibited increased signal (n = 2) with sham animals on PID 3 indicates statistical significance (*t*-test, *p* = 0.0001). There were no statistically significant differences between PID 1 and 5. The timing of peak viremia on PID 3 is in general agreement with the time of peak viremia measured using qRT-PCR in the serum of animals inoculated with ZIKV PRVABC59 [16].

## 4. Discussion

Prior to being tested in human subjects, potential antiviral treatments for ZIKV, including antibodies, should be assessed in vitro and in vivo for activity and potency. The use of reporter viruses can aid in performing such studies, given their potential for use in high-throughput assays.

We produced and characterized two ZIKV reporter viruses, one expressing the original nanoluciferase, nLuc [9] and the other a version where we replaced nLuc with the teLuc enzyme variant. The teLuc contains three amino acid substitutions in the active site (D19S, D85N and C164H) and is named for the peak emission in the teal color range (wavelength 502 nm) [11]. Both nLuc- and teLuc-ZIKV were infectious to cell lines susceptible to other ZIKV isolates (Figure 2 and Figure 3). Interestingly, although the teLuc enzyme is reported to perform best with a specially designed substrate [11], we found that it performed well with the NanoGlo kit (Promega) which contains furimazine substrate. A side-by-side comparison of the nLuc and teLuc activities in three cell lines infected with the respective viruses (Figure 3) showed similar signals. Thus, the data indicate that both nLuc- and teLuc-ZIKV are well suited for in vitro assays.

The nLuc-ZIKV reporter virus was tested in several assays to demonstrate its utility and ease of use, including neutralization assays for two monoclonal antibodies: mAb14 anti-ZIKV antibody and mAb17 anti-flavivirus antibody (Figure 4a), using bioluminescence as a readout. Plotting the relative bioluminescence versus antibody concentration results in similar graphs as the neutralization curves measured using PRVABC59 [12]. Thus, using ZIKV reporter virus to perform neutralization assays can be useful in identifying neutralizing versus non-neutralizing antibodies, and, for the former, concentrations resulting in 50% and 90% inhibition of viral infection (IC_50_ and IC_90_, respectively). These are important parameters to consider when screening plasma donors, antibodies, or even other antiviral candidates for pharmaceutical development. Furthermore, having these infectious clones enables facile manipulations including assessment of clinical/circulating mutations that could enable escape from existing immunity or antiviral therapy. Combining reporter genes with replication incompetent viral constructs is another approach that has been used for neutralization assays of ZIKV and other flaviviruses [17,18,19] with the added benefit of improved biosafety, an important consideration for highly pathogenic viruses.

In previous studies, we have shown that MDCK cells that overexpress the FcRn receptor are more susceptible to infection with the ZIKV PRVABC59 strain than those that do not [12]. FcRn is a ubiquitous receptor important for maintaining IgG homeostasis as well as mediating IgG placental passage from the maternal to the fetal circulation during gestation [20], and has been shown to play a role in viral entry and lifecycle [21,22]. Using the nLuc-ZIKV reporter virus, we reproduced our previous observation (Figure 4b). Furthermore, we found that MDCK/FcRn confluent layers allow for more infectious virus to be transferred from apical to basal side than control cells (Figure 4c). Although not statistically significant, the trend is intriguing and warrants further examination as it could indicate that transcytosis pathways, such as those playing a role in viral transmission of other viruses such as HIV [23], may be implicated. Similarly, we also showed that transfer of infectious virus particles can occur in models of maternal–fetal interface, i.e., confluent layers of trophoblast BeWo and Jeg-3 cell lines grown on semi-permeable membranes (Figure 4d–f). The flowthrough from such placenta models retained the ability to infect Vero (Figure 4d,e) and BeWo (Figure 4f) cells. Importantly, the addition of 10 µg/mL anti-ZIKV neutralizing antibody resulted in a reduction of infectious particles when tested in both Vero and BeWo cells.

It has been shown that anti-ZIKV or anti-flavivirus antibodies, including a hyperimmune polyclonal preparation, can result in the enhancement of infection and disease in cell culture and animal studies, especially at sub-neutralizing concentrations [24,25,26]. In a recent study, we also showed that, depending on the antibody concentration, attenuation of neutralization and even enhancement of infection can be seen in MDCK/FcRn and, to a much smaller extent, BeWo cells [12]. In both of our studies, 10 µg/mL anti-ZIKV antibody did not result in enhancement of infection in BeWo or Vero cells. We believe that adding the transport through placental cells to our infectivity experiments allowed us to model at least one of the processes that plays a major role in infection propagation from maternal to fetal compartments in presence and absence of antibodies. Similar experiments can be performed using multiple antibody concentrations, or other relevant cell types, such as placental macrophage or other immune cells.

Finally, using nLuc-ZIKV reporter to perform an in vivo infection study in immune-competent guinea pigs, we showed that a subset of the guinea pigs inoculated with nLuc-ZIKV displayed increased bioluminescence on PID 3 compared to sham controls (Figure 5). The variability in response depending on viral strain, age, dose, and laboratory where the study was performed has been reported in this species [16,27,28,29] and is also likely the case in the human population. Using these variants in animal models highly susceptible to ZIKV infection, such as mouse knockout strains lacking interferon responses, may provide a more robust model for studying this infection using live imaging.

## 5. Conclusions

The nLuc- and teLuc-ZIKV reporter viruses we produced retain the ability to propagate in various cell lines commonly used for the production and study of this ZIKV. They perform as expected in antibody neutralization assays, and, when coupled with bioluminescence readout, have the potential to facilitate high-throughput assaying of multiple samples, for example, for blood or plasma screening or when assessing potential antibody therapies. Furthermore, they can be useful research tools, including when evaluating the spatial and temporal kinetics of ZIKV infection and treatments in live animals.

## Figures and Tables

**Table 1:** Primers used for cloning.

Name	Sequence	Amplicon Size, Base Pairs
ZIKV-2A-teLuc-Dir	ATGCAATCCCGGGCCCatggtcttcaca	541
teLuc-Ubi-Rev	GAAGATCTGCATcgccagaatgcg	
teLuc-Ubi-Dir	cgcattctggcgATGCAGATCTTC	331
ZIKV-Mlu-Rev	TGAGACCACCgAATGGTGACA	

## Data Availability

The datasets used and/or analyzed during the current study are available from the corresponding author on reasonable request.
